# Low-grade inflammation predicts persistence of depressive symptoms

**DOI:** 10.1007/s00213-015-3919-9

**Published:** 2015-04-16

**Authors:** A. Zalli, O. Jovanova, W. J. G. Hoogendijk, H. Tiemeier, L. A. Carvalho

**Affiliations:** Department of Epidemiology and Public Health, University College London, 1-19 Torrington Place, London, WC1E 6BT UK; Department of Epidemiology, Erasmus MC, University Medical Centre Rotterdam, Rotterdam, The Netherlands; Department of Psychiatry, Erasmus MC, University Medical Centre Rotterdam, Rotterdam, The Netherlands

**Keywords:** Mood disorder, Pro-inflammatory cytokines, Chronic illness, Epidemiological, Psychosomatic, Aging

## Abstract

**Rationale:**

Evidence suggests that depression is cross-sectionally and longitudinally associated with activation of inflammatory response system. A few studies, however, have investigated the longitudinal relationship between raised inflammatory biomarkers and persistence of depressive symptoms. We examined the temporal relationship between serum levels of inflammatory biomarkers and persistence of depressive symptoms among older participants.

**Methods:**

Center for Epidemiologic Studies Depression Scale (CES-D) was used to assess depressive symptoms at baseline and at 5-year follow-up in 656 participants (233 men, 423 women) aged >60 years of the Rotterdam Study. Markers of inflammation interleukin (IL)-6, alpha-1-antichymotrypsin (ACT) and C-reactive protein (CRP) were assessed at baseline, and all participants taking antidepressant medications were excluded from the analysis.

**Results:**

No cross-sectional association was found between IL-6, ACT and CRP with depressive symptoms at baseline. However, higher levels of IL-6 and CRP predicted depressive symptoms at 5-year follow-up. Adjustment for confounding variables had no impact on the observed associations. Similarly, a positive association was found between baseline levels of IL-6 (OR = 2.44, *p* = 0.030) and CRP (OR = 1.81, *p* = 0.052) and persistence of depressive symptoms over 5 years.

**Conclusion:**

Our data suggest that dysregulation of the inflammatory response system is associated with a more severe form of depression more likely to re-occur.

## Introduction

There is evidence implicating inflammation as a potential etiologic factor for mood disorders. Meta-analysis studies have reported higher levels of inflammatory cytokines (such as interleukin-6, (IL-6)) and acute phase proteins (such as C-reactive protein (CRP)) in the peripheral blood and cerebrospinal fluid of patients with major depression (Dowlati et al. 2010; Howren et al. 2009). In addition, inflammation and depressive symptoms seem to be associated in large epidemiological cross-sectional studies (Liu et al. 2014; Matthews et al. 2010; van den Biggelaar et al. 2007).

Further evidence stems from prospective studies showing that acute or chronic administration of cytokines leads to development of depressive symptoms. Chronic administration of the pro-inflammatory cytokine interferon-alpha (IFN-α) for treatment of hepatitis C induced clinically significant depression in 30–50 % of persons with no psychiatric disorders previous to interferon-alpha (IFN-α) treatment (Alavi et al. 2012; Birerdinc et al. 2012). Additional evidence to support an association between elevated immune-inflammatory cytokines and depressive-like behavioural systems is provided in animal models. Elevated immune-inflammatory cytokines induce and exacerbate depressive-like symptoms, whereas tumour necrosis factor-alpha (TNF)-α and IL-6 receptor knockout mice show reduced behavioural indices of depression (Chourbaji et al. 2006; Dantzer 2001; Dantzer et al. 2008; Felger et al. 2007; McNamara and Lotrich 2012; Simen et al. 2006). In humans, fewer large epidemiological studies point towards a possible association between activation of inflammatory system and future depressive symptoms (Valkanova et al. 2013; van den Biggelaar et al. 2007; Wium-Andersen et al. 2013), although controversy still exists (Stewart et al. 2009).

A possible explanation for this controversy is the hypothesis that activation of the inflammatory system distinguishes a particular subset of patients, i.e. those who have a more severe form of depression. In agreement, an activation of the inflammatory system was particularly observed in major depressive patients who are older (Grosse et al. 2014); have recurrent episodes (Ford and Erlinger 2004; Grosse et al. 2014); have comorbid depression with other mental and physical illnesses (Jones et al. 2004; Liukkonen et al. 2011; Moussavi et al. 2007); present earlier onset of the disorder (Grosse et al. 2014) and are resistant to antidepressant treatment (Carvalho et al. 2008, 2014). Consistent with the hypothesis that inflammation is present in a particular subgroup of depressed patients, the anti-inflammatory drug infliximab showed antidepressant properties only in treatment-resistant depressed patients who have high levels of the inflammatory marker C-reactive protein (Raison et al. 2013).

In this study, we take a longitudinal approach to examine the temporal relationship between the inflammatory biomarkers IL-6, alpha-1-antichymotrypsin (ACT) and CRP at baseline and incident depressive symptoms 5 years later or persistent depressive symptoms over 5 years. Participants taking antidepressant medications were excluded from the analysis due to the small population size.

## Methods

### Study population

**Fig. 1:**
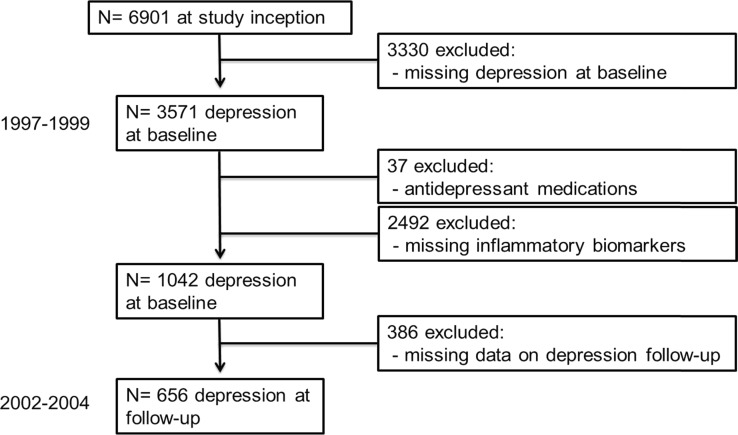
Flow chart for sample selection in the study

The present study is embedded within the Rotterdam Study, a population-based cohort study in which all inhabitants age 55 and over living in a defined geographic area of Rotterdam have been invited to participate (Hofman et al. 1991). The Medical Ethics Committee approved the study according to the Wet Bevolkingsonderzoek: ERGO (Population Study Act: Rotterdam Study) executed by the Ministry of Health, Welfare and Sports of the Netherlands. Written informed consent was obtained from all participants. During the third survey (1997–1999), participants were assessed for depressive symptoms. For the present study, of the 3571 persons who were screened for depressive symptoms, only 1211 had available information on serum level of inflammatory biomarkers. Moreover, 37 participants were excluded for being on antidepressants, leaving 1175 participants eligible for analysis. In the present analysis, we studied the association of inflammatory proteins and depressive symptoms in 656 participants free of antidepressant medications. Out of these participants, 102 were on anti-inflammatory medications. In this study, we do not have repeated measures of inflammatory cytokines (IL-6, ACT and CRP) and therefore could not analyse whether long-term exposure to high levels of inflammatory markers are associated with depression. Sequential exclusions occurred according to the flow chart represented in Fig. 1.

### Depression assessment

Depressive symptoms were assessed through participant’s completion of the Dutch version of the original Center for Epidemiological Studies Depression Scale (CES-D) during a home interview (Beekman et al. 1997; Luijendijk et al. 2008). The CES-D is a self-reporting 20-item measure of depressive symptoms scored on a scale from 0 to 3. For the analysis of persistent depressive symptoms, we used a score of 16 as a cut-off, to indicate clinically significant depressive symptoms in each wave (Beekman et al. 1997; Blazer et al. 1991). Persistent depressive symptoms were defined by having CES-D scores ≥16 in 1997–1999 (baseline) and when re-assessed in 2002–2004 (follow-up).

### Blood specimens

At baseline, a venipuncture was performed by application of minimal stasis with a 21-gauge butterfly needle with tube (Surflo winged infusion set, Terumo). Fasting blood was collected in the morning, and all tubes were put on ice directly and centrifuged at 2000 × *g* for 10 min. Plasma was separated and dispensed into two 1.5-mL aliquots and then frozen within 3 h at −80 °C. Both ACT and CRP were assessed by means of a nephelometric method (BN 100, Dade Behring, Marburg, Germany). The IL-6 concentrations were determined with quantitative enzyme-linked immunosorbent assay with a test kit from R&D systems (Minneapolis, MN). The intra-assay and inter-assay coefficients for all measurements were <5 and <8 %, respectively. High-sensitivity CRP was measured in a serum, which was stored at −20 °C until performance of the CRP measurements, using a rate near-infrared particle immunoassay (IMMAGE, Immunochemistry System, Beckman Coulter, San Diego, CA, detection limit 0.2 mg/L, coefficient of variation (CV) 3.2 %). In this matter, a fully automated Hitachi 747 system (Hitachi, Tokyo, Japan, detection limit 1 mg/L, CV <5 %) was used. IL-6 plasma levels were determined using a quantitative enzyme-linked immunosorbent assay (ELISA) technique (Quantikine HS IL-6 kit, R&D Systems, Oxon, UK, detection limit 0.094 pg/mL, CV 8.7 %) and ACT plasma levels using kinetic nephelometry (Behring Nephelometer BN200, Marburg, Germany, detection limit 1.5 mg/dL, CV 2.8 %).

### Other measurements

The following variables were considered as possible confounding variables: age, sex, education (low, middle, high), physical illness (including prevalent stroke, cardiovascular disease and diabetes), cognitive function (as measured by the mini-mental state examination), smoking and body mass index (BMI). A history of stroke was obtained through direct questioning and computerized linkage with general practitioner medical records (Ikram et al. 2008). Smoking was coded as number of cigarettes currently smoked per day and in categories of current, former and never smoker. To exclude for obvious signs of inflammation when we analysed IL-6 and ACT, we adjusted for acute inflammation as defined by C-reactive protein level >10 mg/L. BMI was defined by >18.50 kg/m^2^ underweight, 18.50–24.99 kg/m^2^ normal, 25–29.99 kg/m^2^ overweight and >30 kg/m^2^ obese.

### Statistical analysis

All data analysis was performed with IBM SPSS Statistics 22. We normalized the distribution of IL-6, ACT and CRP by natural logarithmic transformation and used binary logistic regression to estimate the odds ratio (OR) and 95 % confidence intervals (CIs) of inflammatory markers for depressive symptoms at baseline (1997–1999). To assess the association between the log of the mean IL-6, ACT, CRP and depressive symptoms at 5-year follow-up, we performed linear regression analysis of the CES-D scores at 5-year follow-up (2002–2004) with adjustment for depressive symptoms at baseline (1997–1999). To further explore the association between inflammatory proteins and persistent depressive symptoms over the 5 years (1997/1999–2002/2004), multinomial binary logistic analysis were performed using the CES-D scores (≥16) at baseline and at 5-year follow-up.

Age (continuous) and sex were controlled for in all analyses. To further analyse the effect of confounding factors, we added potential confounders to the basic model. If this changed the effect estimate meaningfully, the contribution of each variable was individually explored. In subgroup analyses, we assessed the age- and sex-adjusted association following exclusion of participants with acute inflammation or those with low mini-mental state examination (MMSE) scores.

## Results

### Demographic characteristics

Table 1 represents information on socio-demographic and clinical baseline characteristics of the participants. The average age of the study participants was 73 years (range 61.1–105.8); 59.9 % of whom were women. Among the study participants, the majority was either classified as being overweight or obese (65.3 %). Most participants were past smokers 48.4 %. Only the minority of participants reported history of physical illness (34 %) or were screened positive for cognitive impairment (13.7 %). Mean scores of levels of pro-inflammatory biomarkers in study participants are reported.

**Table 1:** Demographic characteristics of participants in Rotterdam Study

Demographics		Characteristics
Age (years) (mean, range)		73 (61–106)
Gender (%)		
	Male	40.1
	Female	59.9
Education (%)	Low	61.2
	Middle	29.1
	High	9.7
BMI (kg/m^2^)		
	>18.50	0.9
	18.50–24.99	33.8
	25–29.99	46.4
	30–40	18.9
Smoking status (%)		
	Non-smoker	35.2
	Past smoker	48.4
	Current smoker	16.4
History of physical illness (%)		34
MMSE score (%)		
	>26	13.7
	≤26	86.3
Interleukin-6 (pg/mL)		4.07 (0.53–80)
α1-Antichymotrypsin (mg/dL)		40.3 (19.5–128.5)
C-Reactive protein (mg/L)		3.39 (0.06–88.8)

*BMI* body mass index, *physical illness* stroke, history of cardiovascular disease and diabetes, *MMSE* mini-mental state examination, *IL*-*6* interleukin-6, *ACT* α1-antichymotrypsin, *CRP* C-reactive protein

### No association of IL-6, ACT or CRP and depressive symptoms at baseline

To determine if there was a cross-sectional relationship between serum levels of inflammatory biomarkers IL-6, ACT or CRP and current depressive symptoms at baseline, we performed logistic regression analysis with CES-D scores at baseline. Our data indicate no association between inflammatory proteins IL-6 (OR = 1.08; *p* = 0.731), ACT (OR = 1.069; *p* = 0.670) and CRP (OR = 0.851; *p* = 0.819) and current depressive symptoms at baseline following adjustment for age and gender.

### IL-6 and CRP predict depressive symptoms at 5-year follow-up in older people

To investigate if inflammatory markers are predictive of depressive symptoms at 5-year follow-up, stepwise linear regression were performed with the CES-D scores at 5-year follow-up (Table 2). In our basic model, we corrected for age, gender and depressive symptoms at baseline. Subsequently, corrections included acute inflammation, socio-demographic and health characteristics. Our data indicate that IL-6 (*B* = 0.084, *p* = 0.016) and CRP (*B* = 0.086, *p* = 0.013) were significant predictors of depressive symptoms at 5-year follow-up and remained so after correction for all socio-demographic and health characteristics. ACT (*B* = 0.057, *p* = 0.083) showed a trend association with depressive symptoms at 5-year follow-up, following adjustment for age, gender and depressive symptoms at baseline. The trend association for ACT disappeared after correcting for additional socio-demographic and health characteristics including BMI, smoking, physical illness, low MMSE scores and acute inflammation (Table 2).

**Table 2:** The association between inflammatory proteins and depressive symptoms after 5 years

Inflammatory proteins	No. of cases	*B* (95 % CI)	*P* value
Log IL-6 (per 1 SD increment)α			
Model 1	656	0.107 (1.32, 5.38)	0.001
Model 2	650	0.105 (1.16, 5.33)	0.002
Model 3	650	0.084 (0.48, 4.73)	0.016
Log ACT (per 1 SD increment)α			
Model 1	655	0.057 (−0.67, 10.76)	0.083
Model 2	650	0.048 (−1.57, 10.04)	0.153
Model 3	650	0.020 (−4.20, 7.75)	0.561
Log CRP (per 1 SD increment)α			
Model 1	656	0.090 (0.55, 3.19)	0.006
Model 2	650	0.086 (0.38, 3.16)	0.013

The category “depressive symptoms” includes all subjects who were screened positive. α = levels of inflammatory biomarkers from measurements of 1997–1999: new onset of depressive symptoms at 5-year follow-up in participants with no depressive symptoms at baseline. Subjects on antidepressant medications were excluded from the analysis. *Model 1* linear regression analysis adjusted for age and gender. *Model 2* as model 1 and additionally adjusted for body mass index (BMI), smoking, physical illness (including stroke, history of cardiovascular disease and diabetes) and mini-mental state examination (MMSE). *Model 3* as model 2 and additionally adjusted for acute inflammation. No model 3 was created for CRP as acute inflammation was calculated as CRP >10 mg/mL; therefore, no adjustment for acute inflammation could be done when CRP was used as a predictor

*IL*-*6* interleukin-6, *ACT* α1-antichymotrypsin, *CRP* C-reactive protein, *SD* standard deviation, *B* standardized beta, *CI* confidence interval

### The contribution of individual covariates in the association between inflammatory markers and depressive symptoms at 5-year follow-up

#### Association with IL-6 or CRP

In the secondary analysis, we included adjustments for individual covariates to further assess the effect of each variable on the association between inflammatory markers at baseline and depressive symptoms at 5-year follow-up (Tables 3 and 4). Our results indicate that the association between the levels of IL-6 or CRP and depressive symptoms remained strong and was not substantially affected by age, gender, BMI, smoking status, physical illness, low MMSE scores or acute inflammation (specific to IL-6 analysis only), when adjusted individually or in combination. As acute inflammation was calculated as CRP >10 mg/mL, no adjustment for acute inflammation could be done when CRP was used as a predictor.

**Table 3:** The association between levels of IL-6 and depressive symptoms after 5 years

Inflammatory proteins	No. of cases	*B* (95 % CI)	*P* value
Log IL-6 (per 1 SD increment)α			
Age, sex	656	0.107 (1.32, 5.38)	0.001
Age, sex, BMI (kg/m^2^)	652	0.109 (1.31, 5.46)	0.001
Age, sex, smoking status	656	0.105 (1.24, 5.32)	0.002
Age, sex, physical illness	656	0.106 (1.26, 5.34)	0.002
Age, sex, MMSE	654	0.106 (1.27, 5.33)	0.002
Age, sex, acute inflammation	656	0.088 (0.68, 4.83)	0.009
Fully adjusted*	650	0.084 (0.48, 4.73)	0.016
Subgroups (age and sex adjusted)			
Excluding those with acute inflammation	627	0.089 (0.68, 4.91)	0.010
Excluding those with MMSE	416	0.170 (2.99, 8.12)	0.001

Individual contribution of each covariate

The category “depressive symptoms” includes all subjects who were screened positive. *α* = levels of IL-6 from measurements of 1997–1999: new onset of depressive symptoms at 5-year follow-up in participants with no depressive symptoms at baseline. Subjects on antidepressant medications were excluded from the analysis

*IL*-*6* interleukin-6, *SD* standard deviation, *B* standardized beta, *CI* confidence interval, *BMI* body mass index, *MMSE* mini-mental state examination

In the IL-6 subgroup, exclusion of participants with acute inflammation (*B* = 0.089, *p* = 0.010) or low MMSE scores (*B* = 0.170, *p* = 0.001) did not alter the significance of the association between levels of IL-6 and depressive symptoms at 5-year follow-up (Table 3).

Similarly, in the fully adjusted model, IL-6 predicted incident depressive symptoms after 5 years following exclusion of CRP values >10 mg/L (full model: *B* = 0.089, *p* = 0.013, *n* = 622).

In contrast to IL-6, the association between levels of CRP and incident depressive symptoms was no longer observed (full model: *B* = 0.044, *p* = 0.223, *n* = 622) following exclusion of subjects with CRP values >10 mg/L and adjustment of all covariates.

Similarly to IL-6, in the CRP subgroup, exclusion of participants with low MMSE scores (*B* = 0.108, *p* = 0.008) did not affect the association between CRP levels and depressive symptoms (Table 4). Our data reported no significant gender and IL-6 or CRP interaction in the prediction of depressive symptoms at 5-year follow-up (following adjustment for age); thus, no further analysis stratified for gender was performed.

**Table 4:** The association between levels of CRP and depressive symptoms after 5 years

Inflammatory proteins	No. of cases	*B* (95 % CI)	*P* value
Log CRP (per 1 SD increment)α			
Age, sex	656	0.090 (0.55, 3.19)	0.006
Age, sex, BMI (kg/m^2^)	652	0.090 (0.46, 3.23)	0.009
Age, sex, smoking status	656	0.088 (0.50, 3.15)	0.007
Age, sex, physical illness	656	0.089 (0.51, 3.16)	0.007
Age, sex, MMSE	654	0.089 (0.53, 3.16)	0.006
Fully adjusted*	650	0.086 (0.38, 3.16)	0.013
Subgroups (age and sex adjusted)			
Excluding those with MMSE	416	0.108 (0.63, 4.06)	0.008

Individual contribution of each covariate

The category “depressive symptoms” includes all subjects who were screened positive. α = levels of CRP from measurements of 1997–1999: new onset of depressive symptoms at 5-year follow-up in participants with no depressive symptoms at baseline. Subjects on antidepressant medications were excluded from the analysis

*CRP* C-reactive protein, *SD* standard deviation, *B* standardized beta, *CI* confidence interval, *BMI* body mass index, *MMSE* mini-mental state examination

### Association with ACT

As no association was found between ACT and depressive symptoms at 5-year follow-up, no further analysis was conducted.

### IL-6 and CRP are associated with persistent depressive symptoms over 5 years

To investigate whether there was any association between inflammatory proteins and persistent depressive symptoms over 5 years, we generated cut-off scores CES-D ≥16 at baseline and at 5-year follow-up and subsequently performed logistic analysis. In the basic model 1, IL-6 (OR = 2.32, *p* = 0.035) and CRP (OR = 1.79, *p* = 0.043) were positively associated with persistent depressive symptoms over 5 years (Table 5). In model 2, this association remained relatively unchanged after adjusting for age, gender, BMI, smoking status, physical illness and low MMSE scores for IL-6 (OR = 2.44, *p* = 0.03) and CRP (OR = 1.81, *p* = 0.052), respectively. In contrast to the previous observations on the depressive symptoms at 5-year follow-up (incident depression, Table 2), acute inflammation largely explained the association between IL-6 and persistent depressive symptoms in the fully adjusted model 3 (OR = 2.02, *p* = 0.107) (Table 5). Levels of ACT (OR = 1.38, *p* = 0.80) were not positively associated with persistent depressive symptoms following adjustment for age and gender. No gender interaction was found for IL-6, ACT or CRP in the prediction of persistence depressive symptoms over 5 years following adjustment for age; thus, no further analysis stratified for gender was performed.

**Table 5:** The association between inflammatory proteins and persistent depressive symptoms over 5 years

Inflammatory proteins	No. of cases	Odds ratio (95 % CI)	*P* value
Log IL-6			
Model 1	656	2.32 (1.06, 5.06)	0.035
Model 2	650	2.44 (1.09, 5.45)	0.030
Model 3	649	2.02 (0.86, 4.77)	0.107
Log ACT			
Model 1	654	1.38 (0.12, 16.06)	0.80
Model 2	649	1.23 (0.09, 15.27)	0.871
Model 3	649	0.63 (0.045, 8.81)	0.729
Log CRP			
Model 1	655	1.79 (1.02, 3.13)	0.043
Model 2	649	1.81 (0.99, 3.29)	0.052

The category “depressive symptoms” includes all subjects who were screened positive: CES-D ≥16 at baseline and at 5-year follow-up. Subjects on antidepressant medications were excluded from the analysis. *Model 1* multinomial binary logistic regression analysis adjusted for age and gender. *Model 2* as model 1 and additionally adjusted for body mass index (BMI), smoking, physical illness (including stroke, history of cardiovascular disease and diabetes) and mini-mental state examination (MMSE). *Model 3* as model 2 and additionally adjusted for acute inflammation. No model 3 was created for CRP as acute inflammation was calculated as CRP > 10 mg/mL; therefore, no adjustment for acute inflammation could be done when CRP was used as a predictor

*IL*-*6* interleukin-6, *ACT* α1-antichymotrypsin, *CRP* C-reactive protein, *CI* confidence interval

### The contribution of individual covariates on the association between levels of IL-6 or CRP and persistent depressive symptoms over 5 years

#### Association with IL-6 or CRP

We further investigated whether the individual covariates could explain the correlation between IL-6 or CRP and persistent depressive symptoms during the 5 years (Tables 6 and 7). Our results indicate that the association between the levels of IL-6 and persistent depressive symptoms was little explained by age, gender, BMI, smoking status, physical illness or MMSE, when adjusted individually. In contrast, acute inflammation largely explained the association between the levels of IL-6 and persistent depressive symptoms. Indeed correcting for (OR = 1.96, *p* = 0.113) or excluding (OR = 2.19, *p* = 0.068) participants with acute inflammation reduced this association to insignificant levels (Table 6).

**Table 6:** The association between levels of IL-6 and persistent depressive symptoms over 5 years

Inflammatory proteins	No. of cases	Odds ratio (95 % CI)	*P* value
Log IL-6			
Age, sex	656	2.32 (1.06, 5.06)	0.035
Age, sex, BMI (kg/m^2^)	652	2.54 (1.15, 5.60)	0.021
Age, sex, smoking status	656	2.28 (1.04, 5.00)	0.040
Age, sex, physical illness	656	2.23 (1.01, 4.93)	0.048
Age, sex, MMSE	654	2.38 (1.09, 5.18)	0.030
Age, sex, acute inflammation	655	1.96 (0.85, 4.50)	0.113
Fully adjusted*	649	2.02 (0.86, 4.77)	0.107
Subgroups (age and sex adjusted)			
Excluding those with acute inflammation	626	2.19 (0.94, 5.01)	0.068
Excluding those with MMSE	416	2.64 (1.02, 6.88)	0.046

Individual contribution of each covariate

The category “depressive symptoms” includes all subjects who were screened positive: CES-D ≥16 at baseline and at 5-year follow-up. Subjects on antidepressant medications were excluded from the analysis

*IL*-*6* interleukin-6, *CI* confidence interval, *BMI* body mass index, *MMSE* mini-mental state examination

**Table 7:** The association between levels of CRP and persistent depressive symptoms over 5 years

Inflammatory proteins	No. of cases	Odds ratio (95 % CI)	*P* value
Log CRP			
Age, sex	655	1.79 (1.02, 3.13)	0.043
Age, sex, BMI (kg/m^2^)	651	1.93 (1.07, 3.49)	0.03
Age, sex, smoking status	655	1.76 (1.01, 3.09)	0.048
Age, sex, physical illness	655	1.70 (0.97, 2.98)	0.067
Age, sex, MMSE	653	1.80 (1.02, 3.16)	0.042
Fully adjusted*	649	1.81 (0.99, 3.29)	0.052
Subgroups (age and sex adjusted)			
Excluding those with MMSE	415	1.68 (0.85, 3.35)	0.138

Individual contribution of each covariate

The category “depressive symptoms” includes all subjects who were screened positive: CES-D ≥16 at baseline and at 5-year follow-up. Subjects on antidepressant medications were excluded from the analysis

*CRP* C-reactive protein, *CI* confidence interval, *BMI* body mass index, *MMSE* mini-mental state examination

However, IL-6 predicted persistent depressive symptoms following exclusion of CRP values >10 mg/L and adjustment of all covariates (full model: OR = 2.37, *p* = 0.049, *n* = 622).

In contrast, the association between CRP levels and persistent depressive symptoms was no longer observed following exclusion of subjects with CRP values >10 mg/L (full model: OR = 1.45, *p* = 0.310, *n* = 621). The data suggest that the association between depression and inflammation seems to be stronger for IL-6 than CRP.

In the subgroup analysis, exclusion of participants with low MMSE scores (OR = 2.64, *p* = 0.046) did not affect the positive relationship between levels of IL-6 and persistent depressive symptoms (Table 6). In contrast to IL-6, the association between levels of CRP and persistent depressive symptoms over 5 years was largely explained by adjustment for physical illness (OR = 1.70, *p* = 0.067) and by exclusion (OR = 1.68, *p* = 0.138) but not adjustment for low MMSE scores (OR = 1.80, *p* = 0.042) (Table 7). Association between CRP and depressive symptoms was little explained by age, gender, BMI or smoking status.

#### Association with ACT

As no association was found between ACT and persistent depressive symptoms over 5 years, no further analysis were conducted.

## Discussion

In this large population-based study of elderly persons, we found that the inflammatory biomarkers IL-6 and CRP were longitudinally associated with depressive symptoms. Both IL-6 and CRP levels predicted persistent depressive symptoms over 5 years independently of age, gender, BMI, smoking status or MMSE. Furthermore, we found that IL-6 and CRP predicted incident depressive symptoms at 5-year follow-up after adjustment for all socio-demographic and health characteristics. In contrast, no longitudinal association was observed between the levels of ACT and depressive symptoms. None of the inflammatory biomarkers were associated with depressive symptoms at baseline in the cross-sectional analysis.

To the best of our knowledge, this is the first population-based study to investigate whether inflammation predicts persistent depressive symptoms over 5 years. Persistent depressive symptoms and inflammation had been previously associated in a small study in individuals who were at high risk for coronary heart disease (Azar et al. 2012). Our results are consistent with the literature suggesting that activation of inflammatory response system contributes significantly to the maintenance of symptoms of depression and might be more relevant in people who are more severely ill. Inflammation is particularly observed in major depressive patients who are older (Grosse et al. 2014), have comorbid depression with physical (Jones et al. 2004; Moussavi et al. 2007) and mental illnesses (Liukkonen et al. 2011), present recurrent (Ford and Erlinger 2004) or earlier age of onset of the disorder (Grosse et al. 2014) and in those who are resistant to antidepressant treatment (Carvalho et al. 2008, 2014). Consistent with this hypothesis, we found that the association between inflammation and persistent depression is lost when adjusted for physical illnesses. It is possible that inflammation distinguishes a subgroup of depressed patients. In line with this idea, Raison et al. showed that the anti-inflammatory drug infliximab showed antidepressant properties only in treatment-resistant depressed patients who have high levels of the inflammatory marker C-reactive protein (Raison et al. 2013).

Secondly, in this study, we report a positive longitudinal relationship between IL-6 or CRP levels and incident depressive symptoms at 5-year follow-up. Our data is consistent with findings from previous literature, which demonstrated a positive longitudinal association between inflammation and incident mental health. Similar to our study, Gimeno et al. found a positive association between CRP, IL-6 and incident depressive symptoms in healthy participants (Gimeno et al. 2009). However, in their research, only cognitive depressive symptoms were reported, and mental health was conducted by using the general health questionnaire (GHQ) which is less specific for depressive symptoms. In our study, depressive symptoms were measured using the CES-D scale which is reported to have an excellent sensitivity (100 %) and specificity (88 %) for major depression in a community-based sample of older subjects (Beekman et al. 1997). Also, in agreement with the present results, Kivimaki et al. showed that persistent elevation of IL-6 levels increases risk of common mental disorder (Kivimaki et al. 2014). Likewise, van den Biggelaar and colleagues showed that baseline levels of CRP significantly predicted incident depression at 5-year follow-up in elderly participants of >85 years old (van den Biggelaar et al. 2007).

Further investigations on the link between inflammation and incident depression have also been conducted in different population groups compared to ours. For example, it has been reported that inflammation also predicts depression in females after an accident (Matheny et al. 2011) and in middle-aged adults (Matthews et al. 2010) but not in younger adults (Deverts et al. 2010; Stewart et al. 2009).

This study does not report a cross-sectional association between inflammatory biomarkers and depressive symptoms at baseline. Our data is consistent with findings from Matsushima et al. who reported no cross-sectional association between baseline values of hsCRP and IL-6 and depressive symptoms in a community-dwelling older participants (Matsushima et al. 2015). Our data is also consistent with that of Krogh et al. who showed no cross-sectional association between hsCRP and IL-6 levels and depressive symptoms in people with depression (Krogh et al. 2014). The fact that no cross-sectional association was found suggests that the response to peripheral infection on producing inflammatory cytokines that act on the brain to cause depression might occur only when this inflammatory stimuli is persistent and might not occur in a normal acute and transitional response of the inflammatory system (Dantzer et al. 2008).

Several mechanisms have been speculated on how inflammation and depression may be associated. Both clinical and experimental data strongly point toward the involvement of the enzyme indoleamine 2,3 dioxygenase in the development of inflammation-associated major depressive disorders (Dantzer et al. 2008). Reduced function of the hypothalamus-pituitary-adrenal (HPA) axis has been observed in the presence of chronic inflammation in severely ill patients with major depression (Carvalho et al. 2008, 2014). Inflammation also induces a reduction in the brain-derived neurotrophic factor (BDNF), which negatively influences neurogenesis and neuroplasticity (Cortese et al. 2011; Felger and Lotrich 2013; Lotrich et al. 2013). Over time, a decrease in neurogenesis could contribute to the reduction in hippocampal volume seen in major depression (Campbell and Macqueen 2004) and cognitive dysfunction (Hoglinger et al. 2004) which is positively correlated with a longer duration of depressive symptoms in the community (Beekman et al. 2002). Furthermore, it has been suggested that pro-inflammatory cytokines can modulate the tryptophan/kynurenine pathway and decrease tryptophan availability for serotonin synthesis (Dantzer et al. 2008; Felger and Lotrich 2013; Raedler 2011).

We did not find a longitudinal association between levels of ACT and depressive symptoms. Our findings seem surprising because ACT is an acute phase serum glycoprotein, which is positively associated with CRP (Schaap et al. 2006). A possible explanation for this could be that ACT is a less sensitive marker for inflammation than CRP, which signals early inflammation when other clinical parameters are yet unchanged, and therefore, the latter reveals early inflammation when other clinical parameters are equivocal (Becking et al. 2013; Chard et al. 1988; Lu et al. 2013).

This study has several strengths. First, the assessment of depressive symptoms at baseline and at 5-year follow-up allowed us to examine the direction of the association between inflammatory biomarkers and depressive symptoms. Furthermore, we controlled for several confounding factors, which can influence the association between inflammatory markers and depression. Several limitations of our study need also to be addressed. First, our study was restricted to older population of men and women, and thus, our findings cannot be generalized for the younger population. A second limitation was the fact that we excluded participants who were on antidepressants, and thus, the findings might be not generalizable for patients more severely ill and with clinical depression. Finally, we do not have repeated measures of inflammatory cytokines (IL-6, ACT and CRP) and therefore could not analyse whether long-term exposure to high levels of inflammatory markers are associated with depression.

To conclude, our findings indicate that inflammatory biomarkers IL-6 and CRP are longitudinally associated with persistence and incident depressive symptoms in older men and women, after adjustment for socio-demographic and health characteristics. Inflammation seems to distinguish a particular group of people who may benefit from preventive therapies.
